# Extract of the Blood Circulation-Promoting Recipe-84 Can Protect Rat Retinas by Inhibiting the β-Catenin Signaling Pathway

**DOI:** 10.3390/ijms19092712

**Published:** 2018-09-11

**Authors:** Qiu-Fang Qin, Min Liu, Gui-Hua Tian, Jian Chen, Yu-Sang Li

**Affiliations:** 1 Department of Pharmacology, School of Pharmaceutical Sciences, South-Central University for Nationalities, Wuhan 430074, China; yierss@126.com (Q.-F.Q.); byd80@163.com (M.L.); 2 Chongqing Center for Drug Evaluation and Certification, Chongqing 400042, China; 3 Key Laboratory of Chinese Internal Medicine of MOE and Beijing, Beijing University of Chinese Medicine, Beijing 100700, China; 4 State Key Laboratory of Transducer Technology, Institute of Electronics, Chinese Academy of Sciences, Beijing 100190, China; chenjian@mail.ie.ac.cn

**Keywords:** EBR-84, retinopathy, NMDA, β-catenin, VEGF

## Abstract

Extract of the Blood Circulation-Promoting Recipe (EBR-84) from the Chinese Herbal medicine “Blood Circulation Promoting Recipe” could retard retinopathy development. This study investigated whether EBR-84 protects retinas by inhibiting the β-catenin pathway using a rat model of retinopathy and a retinal ganglion cell 5 (RGC-5) cell death model. RGC death was induced by either *N*-methyl-d-aspartic acid (NMDA) or TWS119 (an activator of the β-catenin pathway). After the corresponding treatment with EBR-84, RGC death and the protein expression levels of β-catenin, cyclooxygenase-2 (COX-2), and vascular endothelial growth factor (VEGF) in rat retinas were examined. β-Catenin accumulated in the retinal ganglion cell layer (GCL) of NMDA-treated rats. EBR-84 (3.9, 7.8, and 15.6 g/kg) significantly attenuated the NMDA-induced RGC loss accompanying the reduction of β-catenin expression. Moreover, the expression levels of COX-2 and VEGF were decreased by EBR-84 in a dose-dependent manner. For the TWS119-treated rats, EBR-84 also ameliorated RGC loss and lowered the expression levels of β-catenin, COX-2, and VEGF. In vitro, EBR-84 increased the viability of NMDA-treated RGC-5 while decreased β-catenin expression. In conclusion, EBR-84 retarded ratretinopathy, and the β-catenin signaling pathway played an important role during this protective process.

## 1. Introduction

High concentrations of *N*-methyl-d-aspartic acid (NMDA) by intravitreal injection cause the loss of retinal ganglion cells (RGCs) and vascular degeneration in rats [1,2,3]. Several signaling proteins participate in the NMDA-induced-RGC loss, including: (1) heat shock proteins [4]; (2) α-amino-3-hydroxy-5-methyl-4-isoxazolepropionic acid (AMPA) [5,6]; (3) transforming growth factor-β (TGF-β) [7]; (4) cyclin-dependent kinase 5 [8]; and (5) the β-catenin signaling pathway. Previous studies indicated that *N*-methyl-d-aspartic acid receptor (NMDAR) activation leads to Wingless/Integrated (Wnt) release and nuclear accumulation of β-catenin in primary cortical cultures [9].

The Wnt/β-catenin signaling pathway was found to play an important role in retinopathy [10]. In the canonical Wnt/β-catenin signaling pathway, without Wnt ligands, β-catenin is phosphorylated by a protein complex containing glycogen synthase kinase-3β (GSK-3β) and degraded rapidly to prevent its accumulation. Upon appropriate stimulation, the β-catenin localized in the cytoplasm can be translocated to the nucleus, where it interacts with members of the T-cell factor family for DNA binding, and regulates the expression of target genes including COX-2 [11,12], fibroblast growth factor (FGF) [13], and VEGF [14].

In our previous study, we found that EBR-84, which was extracted from the traditional Chinese compositus “Huo-Xue-Qu-Yu-Fang” (Blood Circulation-Promoting Recipe), could alleviate the NMDA-induced RGC loss [15]. This mixture, composed of, *Panax notoginseng*, *Salvia miltiorrhiza* Bunge, *Herba Epimedii*, and *Chrysanthemum morifolium* Ramatc, was based on the known functions of each herb used in traditional Chinese medicine for a variety of medical purposes, including retinopathy [16].

Despite the various effects of EBR-84 on retinopathy, knowledge of its effect and mechanism on retinopathy is limited. We hypothesized that EBR-84 is protective against NMDA-induced retinopathy. The present study investigated whether EBR-84 could protect NMDA-induced retinopathy by regulating the Wnt/β-catenin signaling pathway. Retina-injured rat models were generated by the intravitreal injection of NMDA (40 nM). By counting the RGC number and measuring the expression levels of β-catenin, COX-2, and VEGF present in the retinas of rats treated with EBR-84, we examined the neural protective effect of EBR-84 on retinal damage in rats. Additionally, the β-catenin signaling pathway was mimicked by intravitreal injection of TWS119 (a GSK-3β inhibitor) to the rats. This could further prove whether EBR-84 protects retinas by regulating the β-catenin signaling pathway.

## 2. Results

### 2.1. Fingerprint Chromatogram of EBR-84 by High Performance Liquid Chromatography (HPLC)

**Figure 1:**
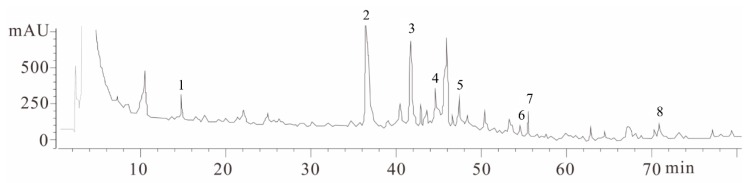
The HPLC fingerprint analysis of the extract from the herbal medicinal compositus prescribed and its main components. The compounds were: Salvianic acid A sodium (1), Salvianolic acid (2), Quercetin-7-*O*-beta-glucoside (3), Rutin (4), Isorhamnetin-3-*O*-beta-glucoside (5), Luteolin (6), Epimedium glucoside (7), and Tanshinone 2A (8).

HPLC chromatograms showed eight marker component peaks present in herbal medicinal compositus prescribed samples and the analyzed peaks had appropriate baseline separations with adjacent peaks. As shown in Figure 1, these components were identified as Salvianic acid A sodium (1), Salvianolic acid (2), Quercetin-7-*O*-beta-glucoside (3), Rutin (4), Isorhamnetin-3-*O*-beta-glucoside (5), Luteolin (6), Epimedium glucoside (7), and Tanshinone 2A (8) by their retention time and UV absorbance of purified standards. To determine the quantity of these eight components in the herbal medicinal compositus prescribe, calibration curves were calculated based on the peak areas obtained from the chromatograms of eight different concentrations of the standard solutions. According to the plot of the peak–area ratio (*y*) vs. concentration (*x*, μg/mL), the regression equations of the eight constituents and their correlation coefficients (*r*) were determined as follows: Salvianic acid A sodium, *y* = 8976*x* − 9488 (*r*^2^ = 0.9995); Salvianolic acid, *y* = 40990*x* − 41287 (*r*^2^ = 0.9996); Quercetin-7-*O*-beta-glucoside, *y* = 61725*x* − 59699 (*r*^2^ = 0.9995); Rutin, *y* = 36799*x* − 38193 (*r*^2^ = 0.9993); Isorhamnetin-3-*O*-beta-glucoside, *y* = 59873*x* − 60198 (*r*^2^ = 0.9992); Luteolin, *y* = 7548.6*x* − 6919 (*r*^2^ = 0.9999); Epimedium glucoside, *y* = 13039*x* − 14678 (*r*^2^ = 0.9998); Tanshinone 2A, *y* = 10128*x* − 11134 (*r*^2^ = 0.9997). The high correlation coefficients of the calibration curves of each concentration indicated a good linearity within the range under investigation. Quantification was performed on the basis of linear calibration plots of the peak areas versus the concentration. The relative contents of components from 1–8 in the herbal medicinal compositus prescribed were 0.67%, 5.87%, 3.79%, 0.76%, 0.89%, 0.95%, 1.69%, and 1.21%, respectively.

### 2.2. EBR-84 Retarded the RGC Loss of the NMDA-Treated Rats

**Figure 2:**
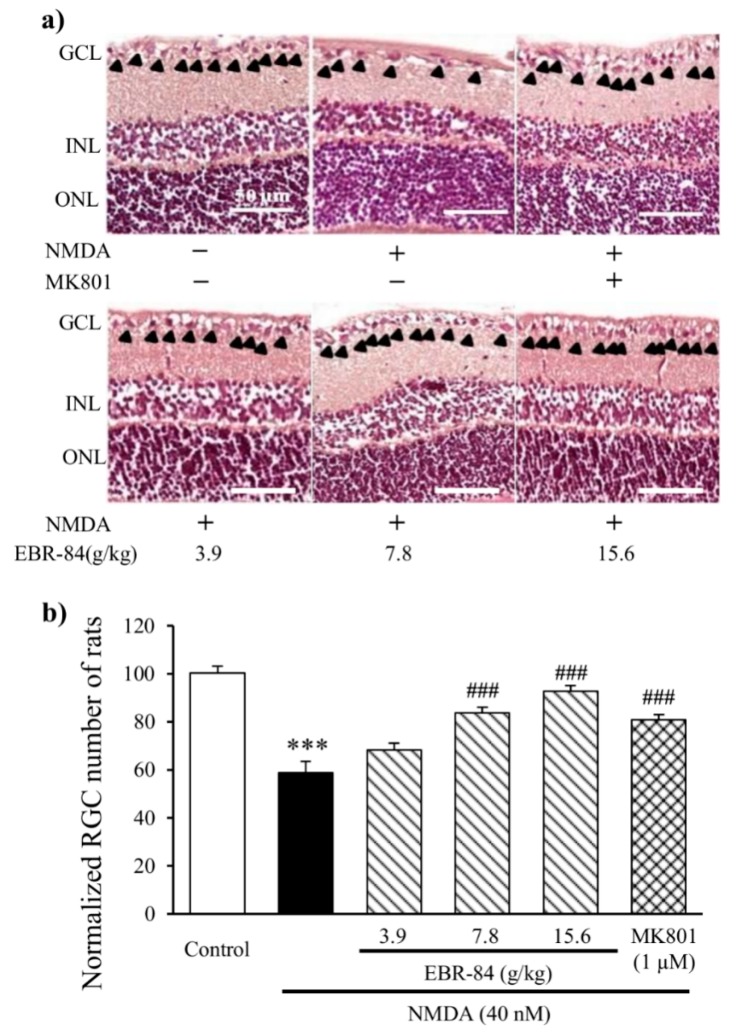
Effect of EBR-84 on the RGCs in NMDA-treated rats. (**a**) Hematoxylin and eosin (H&E) stained staining images of rat retinas. In the control group, the RGCs of the retinas were compact and clear. Obvious RGC loss was found in the NMDA-treated rats. EBR-84 (3.9, 7.8, and 15.6 g/kg) and MK801 (1 μM) notably prevented the loss of the number of RGCs. The black arrows denote RGCs in GCL. (**b**) The normalized RGC numbers in rats (*n* = 5). *** *p* < 0.001 vs. the control group; ^###^ *p* < 0.001 vs. the NMDA group. The magnification of all figures is ×200. Scale bar is 50 μm for all images.

As shown in Figure 2, the number of RGCs in the NMDA-treated rats was 59 ± 5% that of the controls (*p* < 0.001). MK801 is a selective NMDA-receptor antagonist [17,18] that was tested in parallel as a positive control. One micromolar of MK801 was chosen as an effective concentration for this study. As shown in Figure 2b, pretreatment with 1 μM MK801 attenuated the NMDA-induced RGC loss by approximately 22% (*p* < 0.001). Rats were pre-treated for 7 days with EBR-84 (3.9, 7.8, or 15.6 g/kg) before the injection with NMDA, and experiments were continued for another 7 days with persistent EBR-84 treatment. In comparison to the NMDA group, EBR-84 (3.9, 7.8, or 15.6 g/kg) increased the RGC number by approximately 10%, 27%, and 34% (*p* < 0.001), respectively, indicating that pretreatment with EBR-84 could retard RGC loss in a dose-dependent manner. EBR-84 (7.8 g/kg) exhibited a similar effect to MK801 (1 μM).

### 2.3. EBR-84 Decreased Retinal β-Catenin Expression of NMDA-Treated Rats

**Figure 3:**
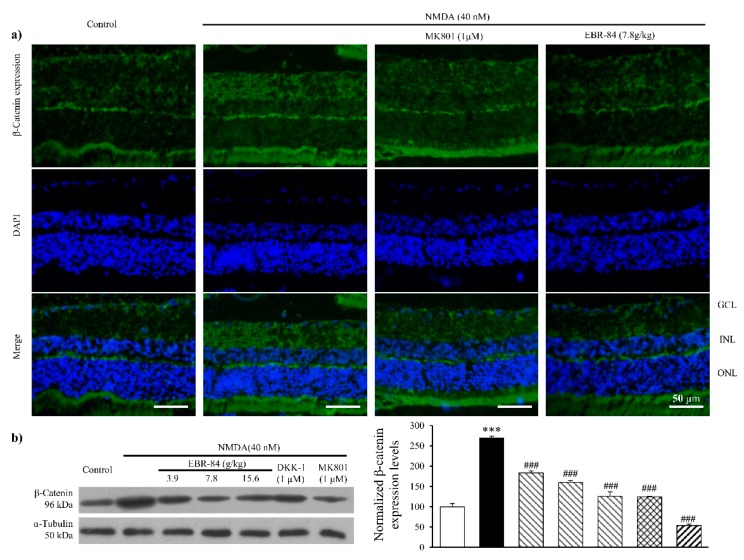
The effect of EBR-84 on β-catenin retinal expression in the RGCL of NMDA-treated rats. (**a**) Representative images of immunofluorescence staining for β-catenin expression in the rat retinas. (**b**) The determination of the nuclear β-catenin protein level in the rat retinas tested using Western blot analysis (*n* = 5). *** *p* < 0.001 vs. the control group; ^###^ *p* < 0.001 vs. the NMDA group. The magnification of all figures is ×200. Scale bar is 50 μm for all images.

The immunofluorescence images show more intensive β-catenin signals in RGCL of NMDA-treated rats compared to normal rats. MK801 and EBR-84 prevented the NMDA-induced RGCL β-catenin expression (Figure 3a). 

A Western blot result revealed a similar increase in β-catenin expression in the NMDA-treated retina (Figure 3b). Pretreatment of rat retinas with Dickkopf-related protein 1 (DKK-1, 50 ng/mL), which is a specific inhibitor of the Wnt pathway, attenuated the NMDA-stimulated retinal β-catenin increase (*p* < 0.001). As shown in Figure 3b, the approximately 2.5-fold increase in the retinal β-catenin level induced by NMDA was significantly inhibited in a dose-dependent manner by pretreatment with EBR-84 at concentrations of 3.7, 7.8, and 15.6 g/kg, which reduced retinal β-catenin by more than 85%, 58%, and 142%, respectively (*p* < 0.001). These results suggested that the protective effect of EBR-84 on the NMDA-damaged retinas might have been due to inhibition of the β-catenin pathway. 

### 2.4. EBR-84 Downregulated COX-2 and VEGF Expression in NMDA-Treated Retinas

**Figure 4:**
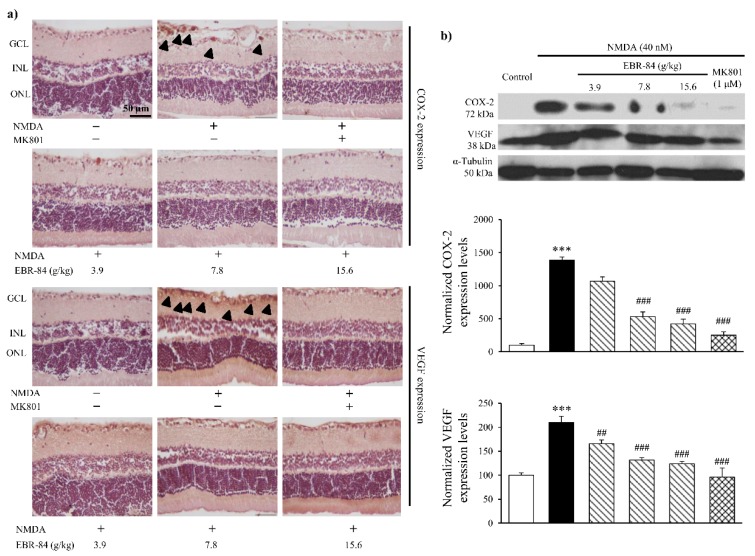
The effects of EBR-84 on the expression levels of COX-2 and VEGF in the retinas of NMDA-treated rats. (**a**) Representative images for immunohistochemical staining of COX-2 and VEGF in the rat retinas. The arrowheads denote the expression of COX-2 or VEGF. (**b**) Quantities of COX-2 and VEGF expression levels using Western blot analysis in the retina of rats (*n* = 5). *** *p* < 0.001 vs. the control group; ^##^ *p* < 0.01 and ^###^ *p* < 0.001 vs. the NMDA group. The magnification of all figures is ×200. Scale bar is 50 μm for all images.

Figure 4a shows COX-2 and VEGF expression in the rat retinas by immunohistochemical assay. Compared to normal rats, although the RGCL neurons in the NMDA-treated rats were badly damaged, markedly elevated COX-2 expression was observed. Not singly but in pairs, in the NMDA-treated rats, the VEGF expression showed a notable increase in RGCL. Pretreatment of EBR-84 (3.9, 7.8, and 15.6 g/kg) and MK801 (1 μM) both prevented the over expression of COX-2 to VEGF in varying degrees.

To further prove the effect of EBR-84 on both COX-2 and VEGF expression, we analyzed their content levels by Western blot assay. As seen in Figure 4b, the approximately 14-fold increase in retinal COX-2 level induced by NMDA was inhibited in a dose-dependent manner by pretreatment with EBR-84 (3.7, 7.8, and 15.6 g/kg). The pretreatment of MK801 (1 μM) greatly attenuated NMDA-stimulated retinal COX-2 increase (*p* < 0.001). There was also a statistically significant difference in the expression of VEGF between the NMDA group and the control group (*p*< 0.001). In addition, the VEGF expression in the RGCL of the rat retinas was dose-dependently reduced by EBR-84 (3.7, 7.8, and 15.6 g/kg) (*p* = 0.006, *p* < 0.001, and *p* < 0.001, respectively).

The results demonstrated that EBR-84 inhibited the NMDA-induced overexpression of COX-2 and VEGF in the RGCL of rats, accompanied by a reduced RGCL β-catenin level, which indicated that ERB-84 could recover the RGCL neuron number by inhibiting the β-catenin pathway.

### 2.5. Effects of EBR on Retinas of TWS119-Treated Rats

**Figure 5:**
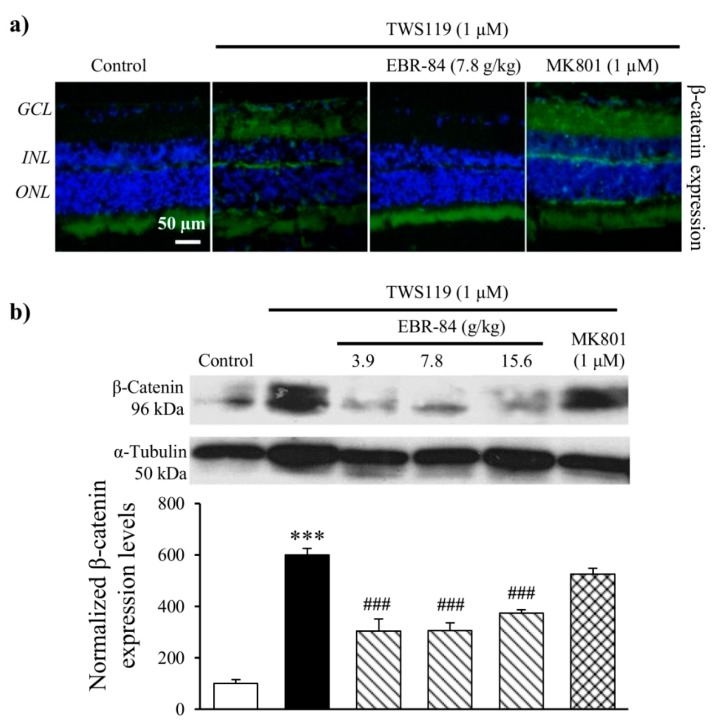
The effects of EBR-84 on β-catenin expression level in the retinas of TWS119-treated rats. (**a**) Representative images of immunofluorescence staining of β-catenin in the rat retinas. (**b**) The determination of nuclear β-catenin protein level from the rat retina tested using Western blot analysis (*n* = 5). *** *p* < 0.001 vs. the control group; ^###^ *p* < 0.001 vs. the TWS119 group. The magnification of all figures is ×200. Scale bar is 50 μm for all images.

To further elucidate whether EBR-84 protects NMDA-damaged retinasby inhibiting β-catenin signaling activation, we injected the rats with 1 μM TWS119, which mimics the canonical β-catenin signaling pathway by inhibiting GSK-3β [19,20]. As shown in Figure 5a, TWS119 led to β-catenin accumulation in the RGCL of rats, while minimal β-catenin accumulation was observed in the RGCL of normal rats. This illustrates that the β-catenin signaling pathway was successfully activated by TWS119 (1 μM). EBR-84 relieved the robust β-catenin accumulation. MK801 (1 μM) showed a limited effect on TWS119-induced overexpression of β-catenin. The inhibitory function was clearly shown by the Western blot result (Figure 5b). β-Catenin expression in the retinas of the EBR-84 (3.9, 7.8 and 15.6 g/kg) treated-rats was only half that of model rats (*p* < 0.001).

**Figure 6:**
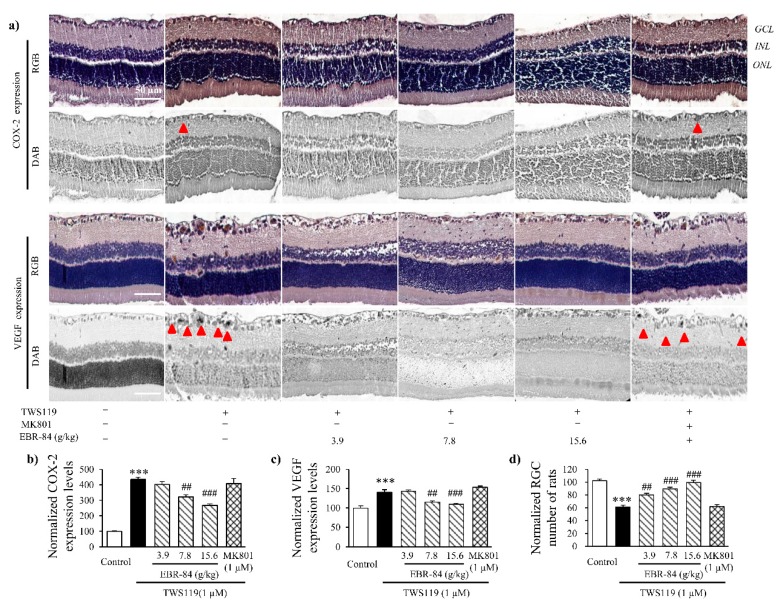
The effects of EBR-84 on COX-2 and VEGF expression and RGC number in the retinas of TWS119-treated rats. (**a**) Representative multispectral images for immunohistochemical staining of COX-2 and VEGF in the rat retinas. The red arrows denote the places that express COX-2 and VEGF in GCL. (**b**) Quantification of normalized COX-2 expression level using multispectral image analysis in the rat retinas (*n* = 5). (**c**) Quantification of normalized VEGF expression level using multispectral image analysis in the rat retinas (*n* = 5). (**d**) The normalized RGC numbersin rats (*n* = 5). *** *p* < 0.001 vs. the control group; ^##^ *p* < 0.01 and ^###^ *p* < 0.001 vs. the TWS119 group. The magnification of all figures is ×200. Scale bar is 50 μm for all images.

COX-2 and VEGF were inspected by immunohistochemical assays, and the expression levels were quantified using a multispectral image analysis (Figure 6a). TWS119 (1 μM) increased the expression level of COX-2 (*p* < 0.001). EBR-84 (7.8 and 15.6 g/kg) decreased the COX-2 expression level by 22% and 44%, respectively (Figure 6b). In Figure 6c, TWS119 increased the VEGF expression level by 3.5-fold. However, EBR-84 (7.8 and 15.6 g/kg) recovered the VEGF expression back to normal levels (*p* < 0.001).

The effect of EBR-84 on the RGCL neuron number of the TWS119-treated rats was then studied. The H&E staining result indicated that EBR-84 (3.9, 7.8, and 15.6 g/kg) remarkably ameliorated the TWS119-caused RGC loss. The cell counting measurement shown in Figure 6d reveals the dose-dependent behavior of EBR-84. Interestingly, MK801 (1 μM) had no obvious effect on either the RGCL neuron number or expression levels of COX-2 and VEGF. These results demonstrated that EBR-84 could protect TWS119-damaged retina by downregulating the β-catenin signaling pathway.

### 2.6. EBR-84 Increased the Viability of NMDA-Treated RGC-5 Cells

**Figure 7:**
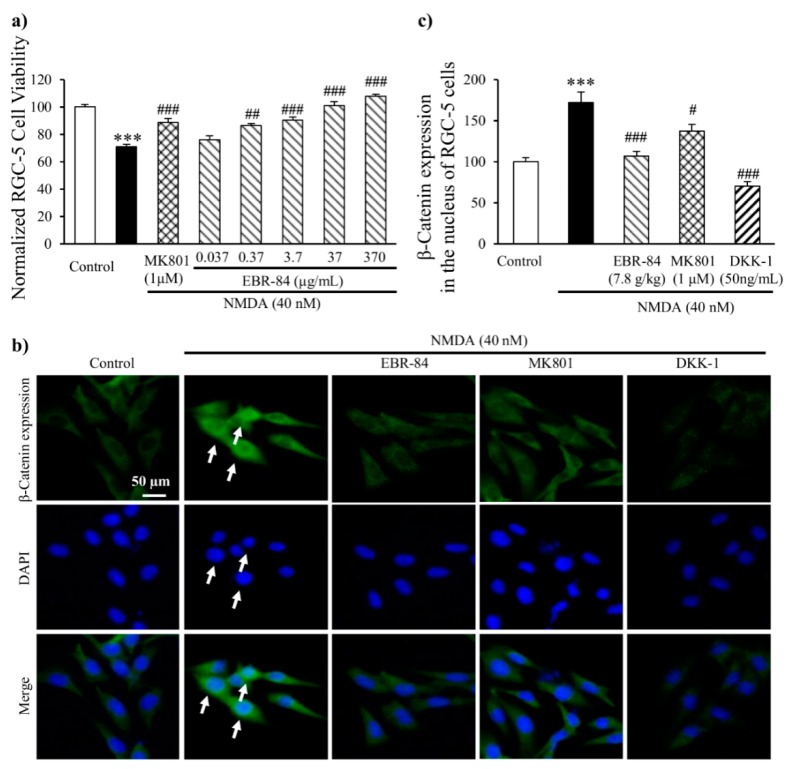
The effect of EBR-84 on cell viability and β-catenin expression of NMDA-treated RGC-5 cells. (**a**) The viability levels of RGC-5 cells in each group were normalized by that of the control group (*n* = 6). *** *p* < 0.001 vs. the control group; ^##^ *p* < 0.01 and ^###^ *p* < 0.001 vs. the NMDA group. (**b**) Representative immunofluorescence staining images for β-catenin expression in the RGC-5 cells. The arrows denote the expression of β-catenin in the nuclei of RGC-5 cells. (**c**) Fluorescence intensity for β-catenin expression in the nuclei of RGC-5 cells. The fluorescence intensity of β-catenin in each group was normalized to that of the control group (*n* = 6). *** *p* < 0.001 vs. the control group; ^#^ *p* < 0.05 and ^###^ *p* < 0.001 vs. the NMDA group. The magnification of all figures is ×200. Scale bar is 50 μm for all images.

The RGC-5 cell line is a transformed proliferating cell line that expresses RGC-specific markers similar to RGCs [21]. First, we found no obvious toxic and deleterious effect of EBR-84 (0.037, 0.37, 3.7, 37, and 370 μg/mL) on RGC-5 cells. Then, we investigated the effect of EBR-84 on the viability of RGC-5 cells incubated with a culture medium including 40 nM NMDA. As shown in Figure 7a, there was a significant difference between the viability of the NMDA group (72.2% of the control) and the control group (*p* < 0.001). As we increased the concentration of EBR-84 (0.037, 0.37, 3.7, 37, and 370 μg/mL), the viability of the RGC-5 cells incubated with NMDA gradually increased. MK801, which is a selective inhibitor of NMDA, showed a similar effect to EBR-84 (3.7 μg/mL).

### 2.7. EBR-84 Depressed the β-Catenin Expression in RGC-5 Cells

We next investigated the effects of EBR-84 on β-catenin expression in the RGC-5 cell line. RGC-5 cells were cultured in Dulbecco’s modified Eagle’s medium (DMEM) with 40 nM NMDA. As shown in Figure 7b, a small amount of β-catenin was expressed in the cytoplasm of the normal retina, but in the NMDA-treated retina, higher β-catenin expression was observed in the nucleus. Moreover, the β-catenin fluorescence intensity in the nucleus of the NMDA group (Figure 7c) showed a 2-fold increase compared with that in the control (*p* < 0.001). EBR-84 (37 μg/mL) and DKK-1 (50 ng/mL) attenuated the NMDA-induced high expression of β-catenin (*p* < 0.001). The depressive effect of 1 μM MK801 on the β-catenin expression was lower than that of the EBR-84 (37 μg/mL) and DKK-1 (50 ng/mL), but still notable (*p* = 0.039). These findings once again demonstrated that EBR-84 blocked the β-catenin pathway, and thereby protected the NMDA-damaged retinas.

## 3. Discussion

Retinal ganglion cells (RGCs) are responsible for the transmission of visual signals to the brain, which plays an important role in retinopathy. Progressive death of RGCs occurring in glaucoma and several other retinal diseases can lead to visual impairment and blindness [18]. Intravitreal injection of rats with NMDA could mimic RGC damage, which may be seen as a model for investigating retinopathy associated with stimulation of NMDA receptors by abnormally high NMDA levels [1]. We found that 40 nM of NMDA could induce the death of RGCs both in vivo and in vitro in this study, and we discussed the protective mechanism of EBR-84 on the RGCs of rats treated with NMDA.

Many signaling pathways are involved in this NMDA-induced RGC damage, including the TGF-β, bone morphogenetic protein [22], and AMPA [5] signaling pathways. The β-catenin signaling pathway gradually became an important part in retinopathy [23]. Abnormal expression of β-catenin was found in the injured retina [24]. Moreover, inhibitors of the β-catenin signaling pathway could alleviate the diabetic retinopathy symptoms in rats [10]. Stimulation of the Wnt/β-catenin signaling pathway may lead to neovascular generation [24].

COX-2 and VEGF are targets of the β-catenin signaling pathway in different tissues [12,14]. COX-2, which is closely related to inflammation, plays a significant role in the production of VEGF and ultimately injures the neurons in the retina [25,26,27]. VEGF is the principal mediator involved in retinal angiogenesis under physiological concentrations [28,29], which leads to vascular degeneration when overexpressed [30]. Takahashi et al. [31] and Sin et al. [32] demonstrated the efficacy of COX inhibition in reducing the production of VEGF and neovascularization. These clues suggest that activation of the β-catenin signaling pathway could lead to retinal RGC loss by upregulating COX-2 and VEGF expressions. In this study, intraperitoneal injection of NMDA (40 nM) elevated retinal β-catenin (an essential effector of the canonical β-catenin pathway), COX-2 and VEGF expression in rats. 

Moreover, we mimicked the β-catenin signaling pathway by intraperitoneal injection of TWS119 (1 μM). Activation of β-catenin signaling triggers a series of molecular events that involves inhibition of the kinase GSK-3β (encoded by GSK-3β), thereby inhibiting the phosphorylation of β-catenin, inhibiting proteasomal degradation, and enhancing accumulation of β-catenin in the cytosol and nucleus. As a result, GSK-3β inhibitors have been used experimentally as pharmacologic activators of Wnt/β-catenin signaling [20]. TWS119, which is a GSK-3β inhibitor, was chosen to induce abnormal β-catenin accumulation in the retina, which mimics the canonical β-catenin signaling pathway [19]. In our recent study, we observed that the peripheral nerve injury-evoked aberrant β-catenin accumulation may promote the induction of COX-2, contributing to the maintenance of nociceptive information [33]. Therefore, the results in this study showed that TWS119 elevated retinal β-catenin, COX-2 and VEGF expression and decreased the number of RGCs in rats.

EBR-84 is an extract of a Chinese herbal blood circulation-promoting recipe, composed of *Panax notoginseng*, *Salvia miltiorrhiza* Bunge, *Herba Epimedii*, and *Chrysanthemum morifolium* Ramatc, etc., which is clinically used to treat retinopathy by cleansing the liver, improving blood circulation, and promoting healing [15,16]. A previous report suggested that Salvianolic acid B from *Salvia miltiorrhiza* Bunge could promote neural stem cell proliferation and differentiation, which may contribute to protecting retinal neurons [34]. Moreover, 3-(3,4-dihydroxyphenyl)-2-hydroxy-propanoic acid and 3,4-dihydroxy-phenyl lactic acid isolated from *Salvia miltiorrhiza* Bunge could ameliorate microvascular disturbance in rat [35,36], which plays a central role in retinal blood circulation modulation. However, the exact protective mechanism of EBR-84 on the damaged retina of rats remains to be elucidated.

In our study, EBR-84 alleviated the NMDA-induced RGC loss. We found that EBR-84 could reduce the overexpression of β-catenin, COX-2 and VEGF in rat retinas. This suggested that EBR-84 protected the retina by regulating the β-catenin signaling pathway. To further elucidate whether EBR-84 protected against retina damaged through inhibiting β-catenin signal activation, we injected the rats with TWS119 (1 μM) to activate the β-catenin signaling pathway. EBR-84 depressed β-catenin expression, downregulated COX-2 and VEGF expression and reversed the neuron loss caused by TWS119. MK801 could inhibit the NMDA-induced retina damage to some extent, because it is a selective NMDA receptor inhibitor, but nevertheless, it could not block the TWS119-induced RGCL neuron loss. Combined with the preceding results, EBR-84 could protect NMDA-damaged retinas by blocking the β-catenin pathway.

In the retina, RGC cells are particularly sensitive to excitotoxicity, and excess glutamate has been proposed to underlie common neurodegenerative disorders of the eye [37]. We also investigated the protective effect of EBR-84 on RGC-5 cells in vitro. The cells incubated with NMDA (40 nM) showed decreasing viability and accumulating β-catenin expression in the nucleus. EBR-84 blocked the nuclear β-catenin accumulation and increased the viability of the RGC-5 cells, the same as the treatment with DKK-1 or MK801. These once again demonstrated that EBR-84 blocked the β-catenin pathway, and thereby protected the RGCs.

In summary, our data show that the expression of β-catenin, COX-2, and VEGF were increased during the RGC loss induced by NMDA or TWS119, while EBR-84 could alleviate the RGC loss by inhibiting the β-catenin signaling pathway. Our findings also suggest that EBR-84 could potentially be developed as effective agents for retinal diseases.

## 4. Materials and Methods

### 4.1. Drug Preparations

*Panax notoginseng*, *Chrysanthemum morifolium* Ramat, *Herba Epimedii*, and *Salvia miltiorrhiza* Bunge were collected from Yunnan, Zhejiang, Sichuan, and Anhui provinces, respectively, in China in 2010 and identified by botanist Hong Zhang (College of Pharmacy, Wuhan University, Wuhan, China). All voucher specimens were deposited at the herbarium of the Department of Pharmacy, South-Central University for Nationalities (Nos. 37, 18, 126, and 80, respectively). These medicinal herbs, which constitute EBR-84, were prepared as followed.

*Panax notoginseng* and *Chrysanthemum morifolium* Ramat were extracted with 10 volumes of 70% ethanol for 90 min at 60 °C. The procedure was repeated twice. The extracts were filtered and then concentrated under vacuum into residues. *Herba Epimedii* and *Salvia miltiorrhiza* Bunge were extracted twice with 15 volumes of hot water (60 °C) for 90 min. After filtration, the supernatant was separated and concentrated. All residues were lyophilized into powder. The EBR-84 was obtained and used for animal administration. The weight ratio of the dried *Panax notoginseng*, *Chrysanthemum morifolium* Ramat, *Herba Epimedii*, and *Salvia miltiorrhiza* Bunge was 1:2:2:2. The respective yields of the extracts were 28%, 31%, 16%, and 19% (*w*/*w*).

### 4.2. Fingerprint Chromatogram of RIE by HPLC

All chromatographic measurements were performed on an Agilent 1200 liquid chromatography system (Agilent, Santa Clara, CA, USA) equipped with avacuum degasser, a quaternary, low-pressure mixing pump, a thermostated column compartment, and an Agilent 1200 photodiode array detector. Chromatographic separation was performed on a Thermo Syncronis RP-C18 column (250 × 4.6 mm, 5 μm, Waltham, MA, USA). The sample solution was filtered through a syringe filter (0.45 μm), and the injection volume was 10 μL. The mobile phase consisted of a 0.1% CF_3_COOH solution (solvent A) and methanol (solvent B) at a flow rate of 1.0 mL/min. The gradient elution program was performed as follows: 0–20 min, 10–30% B; 20–40 min, 30–60% B; 40–60 min, 60–85% B; 60–80 min, 85–100% B, and then back to the stable initial conditions for another 10 min. The column temperature was maintained at 25 °C and the detection wavelength was set at 210 nm for all of the mixtures. The identification of the investigated mixtures was confirmed by comparison of their retention times with those of standards under the same conditions. 

### 4.3. Animal Grouping and Drug Treatment

Experimental male Sprague-Dawley rats (weighing 180–200 g) were provided by Hubei Centers for Disease Control and Prevention (Wuhan, China). Animals were acclimatized to the laboratories for one week prior to manipulation and were group housed in a controlled environment (temperature 20 ± 2 °C, and humidity 70%) under a 12-h light–dark cycle. The rats were allowed free access to food and water. The care and use of animals and experimental protocols for this study were performed according to the Guide for Animal Experimentation, South-Central University for Nationalities and the Committee of Research Facilities for Laboratory Animal Sciences, South-Central University for Nationalities, China. The protocols were approved by the Committee on the Ethics of Animal Experiments of the South-Central University for Nationalities, China (Permit Number: 2013-SCUEC-AEC-006, 10 September 2013).

The protocol of animal grouping and drug treatment is shown in Table 1. EBR-84 was given (intragastric administration) for 14 days to the rats. Seven days from the beginning of drug administration, the rats were anesthetized with an intra-peritoneal injection of 40 mg/kg sodium pentobarbital (Sigma Chemical, St. Louis, MO, USA) and then intravitreally injected with 40 nM NMDA (Sigma Chemical), 1 μΜ MK801 (Sigma Chemical), 1 μM TWS119 (Cayman Chemical, Ann Arbor, MI, USA), or saline with a 33-gauge needle connected to a microsyringe after dilation of the pupil with tropicamide (Sigma Chemical) in both eyes. The total volume was 2 μL. The tip of the needle was inserted through the dorsal limbus of the eye. The rats were sacrificed after the EBR-84 treatment. The left eyecups were enucleated, formalin-fixed for 24 h, and paraffin-embedded for sectioning; the retinas of the right eyecups were dissected from animal eyes and sonicated in cold PBS for Western blot assays.

### 4.4. H&E Staining and Immunohistochemical Analysis

The slides containing the rat eyes were stained with H&E. The RGC number was randomly counted in six areas of the RGC layer (100 μm each) of each section (*n* = 5 per eye) [38]. The immunofluorescence expression levels of β-catenin, COX-2, and VEGF were visualized with an anti-β-catenin polyclonal antibody (1:500, Cayman Chemical, USA), anti-COX-2 polyclonal antibody (1:1000, Cayman Chemical), or anti-VEGF antibody (1:200, Biovision, Milpitas, CA, USA). The tissue sections were then incubated for 1 h at room temperature with Alexa Fluor 488 goat anti-rabbit IgG (1:1000) or Diaminobenzidine (Histofine, Nichirei Corp., Tokyo, Japan), followed by additional washing and then counterstaining with DAPI or hematoxylin. The slides were visualized under a Nikon Eclipse Ti fluorescence microscope (Tokyo, Japan) [15,39].

The multispectral image analysis of slides was referred to our previous study [40]. Briefly, it was performed by using a Nikon 50i light microscope (Nikon) with a Nuance Multispectral Imaging System (Cambridge Research and Instrumentation Inc., Woburn, MA, USA). Spectral optical density data were acquired from 420–720 nm automatically. Spectral unmixing was finished by using Nuance software v1.42 (Woburn, MA, USA) and the pure spectral libraries of individual chromogens. For quantification in each experiment, three equal-sized fields of each photograph per group were randomly chosen.

### 4.5. Western Blot Assay

Western blot analysis was performed as previously described [23]. Anti-β-catenin antibody (1:500), anti-COX-2 antibody (1:1000), or anti-VEGF antibody (1:1000) were used for Western blot analysis. Densitometry was performed using ImageJ software v1.4 (Wayne Rasband, National Institutes of Health, Bethesda, MD, USA) and normalized to α-Tubulin levels [41].

### 4.6. Cell Culture and Viability Assay

RGC-5 cells were purchased from the American Type Culture Collection (Wuhan, China). They were cultured in DMEM containing 10% fetal bovine serum, 25 mM glucose, 100 U/mL penicillin, and 100 μg/mL streptomycin [21]. The cells were maintained in 25 cm^2^ filter-capped cell culture flasks and incubated under 5% CO_2_ at 37 °C. Cells were passaged every 2–3 days. RGC-5 cells of passages 7–15 were used in the present study.

Cell viability of RGCs was assessed using the colorimetric reagent, 3-(4,5-dimethylthiazol-2-yl)-2,5-diphenyltetrazolium bromide (MTT, Sigma Chemical). Briefly, cells were seeded in 96-well plates, with 5000 cells in 100 μL medium per well, and cultured for 24 h for cell stabilization under 5% CO_2_ at 37 °C. Then, the cells were divided into nine groups for a further 24 h culture: control group, NMDA (40 nM), five NMDA+EBR-84 (0.037, 0.37, 3.7, 37, and 370 μg/mL) groups, an NMDA+MK801 (1 μΜ) group, and an NMDA+DKK-1 (50 ng/mL) group. After the treatments with various agents, cells were incubated with MTT (20 μL, 5 mg/mL) as described previously [42]. The initial appropriate concentration of EBR-84 (lower drug concentration and apparent effect) in the RGC-5 cell line was measured.

### 4.7. Immunocytochemical Staining for β-Catenin

Immunocytochemical staining for β-catenin in cultured RGC-5 cells on coverslips was performed as described previously [43]. Briefly, 4% paraformaldehyde-fixed cultured RGC-5 cells on coverslips were incubated with anti-β-catenin polyclonal antibody (1:500, Cayman Chemical, Ann Arbor, MI, USA). They were washed the next day and subsequently exposed for 1 h to Alexa Fluor 488 goat anti-rabbit IgG (1:1000, Molecular Probes, Eugene, OR, USA), followed by additional washing, counterstaining with DAPI, and visualization under a Nikon Eclipse Ti fluorescence microscope [19,44].

### 4.8. Statistical Analysis

The neuron numbers and the expression levels of β-catenin, COX-2, and VEGF in each group were normalized to that of the control group. Data are presented as the means ± Standard error of the mean (SEM). All statistical analyses were performed using the SPSS 19.0 software package (SPSS Inc., Chicago, IL, USA). The statistical significance of differences was determined by a one-way ANOVA with Tukey’s post hoc test. *p* < 0.05 was considered to indicate a significant difference.

## Figures and Tables

**Table 1:** The protocol of animal grouping and drug treatment. (“+” or “−” means NMDA, EBR-84, and MK801 were given or ungiven to the rats).

**NMDA Experimental Group**				
**Groups**	**NMDA (40 nM)**	**EBR-84 (g/kg)**	**MK801 (1 µM)**	**Number of Rats**
Control group	−	−	−	5
NMDA group	+	−	−	5
EBR-84 groups	+	3.9	−	5
	+	7.8	−	5
	+	15.6	−	5
MK801 group	+	−	+	5
**TWS119 Experimental Group**				
**Groups**	**TWS119 (1 µM)**	**EBR-84 (g/kg)**	**MK801 (1 µM)**	**Number of Rats**
Control group	−	−	−	5
TWS119 group	+	−	−	5
EBR-84 groups	+	3.9	−	5
	+	7.8	−	5
	+	15.6	−	5
MK801 group	+	−	+	5

## Abbreviations

AMPA	α-amino-3-hydroxy-5-methyl-4-isoxazolepropionic acid
COX-2	Cyclooxygenase-2
DKK-1	Dickkopf-related protein 1
EBR-84	Extract of the Blood Circulation-Promoting Recipe
FGF	Fibroblast growth factor
GSK-3β	Glycogen synthase kinase-3β
GCL	Ganglion cell layer
H&E	Hematoxylin and eosin
HPLC	High performance liquid chromatography
MTT	3-(4,5-dimethylthiazol-2-yl)-2,5-diphenyltetrazolium bromide
NMDA	*N*-methyl-d-aspartic acid
NMDAR	*N*-methyl-d-aspartic acid receptor
RGCs	Retinal ganglion cells
SEM	Standard error of the mean
TGF-β	Transforming growth factor-β
VEGF	Vascular endothelial growth factor
Wnt	Wingless/Integrated
